# Targeted Next-Generation Sequencing of Liquid Biopsy Samples from Patients with NSCLC

**DOI:** 10.3390/diagnostics11020155

**Published:** 2021-01-21

**Authors:** Hestia Mellert, Jordan Reese, Leisa Jackson, Victoria Maxwell, Chérie Tschida, Gary A. Pestano

**Affiliations:** Biodesix Inc., 2970 Wilderness Place Suite 100, Boulder, CO 80301, USA; jmreese08@gmail.com (J.R.); leisa.jackson@biodesix.com (L.J.); victoria.maxwell@biodesix.com (V.M.); cherie.tschida@biodesix.com (C.T.); gary.pestano@biodesix.com (G.A.P.)

**Keywords:** diagnostics, non-small cell lung cancer (NSCLC), liquid biopsy, next generation sequencing (NGS)

## Abstract

Liquid biopsy tests have become an integral part of the molecular diagnosis of patients with non-small cell lung cancer (NSCLC). We describe a new test panel that uses very low input (20 ng) of cell-free nucleic acids extracted from human plasma, which is designed to yield results in less than 72 h. In this study, we performed novel amplicon-based targeted next-generation sequencing with a semiconductor-based system, the Ion GeneStudio S5 Prime. The analytic performance of the assay was evaluated using contrived and retrospectively collected clinical specimens. The cumulative percent coefficient of variation for the new test process was very precise at 8.4% for inter-day, 4.0% for inter-operator and 3.4% for inter-instrument. We also observed significant agreement (95.7–100%) with an orthogonal, high-sensitivity droplet digital™ Polymerase Chain Reaction (ddPCR) test. This method offers a valuable supplement to assessing targeted mutations from blood while conserving specimens and maintaining sensitivity, with rapid turn-around times to actionable results.

## 1. Introduction

The successful implementation of precision medicine for the treatment of non-small cell lung cancer (NSCLC) relies on well-characterized molecular features of the patients’ tumor. Genotyping of tissue obtained from NSCLC tumors provides actionable information in many cases [1]. However, tumor tissue is not always accessible for molecular testing and, when available, can miss important information because a tissue biopsy does not represent the entirety of the tumor, but rather is a sample of a heterogeneous malignancy [1]. Moreover, as the tumor evolves naturally or from challenge by therapy, the molecular landscape changes, sometimes dramatically, requiring additional molecular testing that is temporally associated with the disease state [2].

Blood-based assays have become increasingly accepted as a supplement to tissue-based testing approaches and may address the inaccessibility of lesions to biopsy, limited tissue quantity, and the invasiveness associated with tissue collection procedures [3,4]. For these reasons, liquid biopsy for profiling tumor-originating nucleic acids has become important in the diagnostic workup for cancer patients, particularly in NSCLC.

Regulatory agencies and physician-led associations have provided guidance for the use of liquid biopsy in clinical practice for directing targeted therapy selection [5,6]. The guidance was initially limited to diagnosis when tissue was not available, but liquid biopsy is now considered a complimentary method to tissue. If the plasma is negative, a tissue biopsy is recommended when possible [7]. This approach leverages the benefits of liquid biopsy while still minimizing the risk of missing actionable variants in those individuals not shedding tumor-derived DNA into their bloodstreams. Sensitivity of liquid biopsy applications when compared to matched tissue are between 70% and 85% in late-stage disease and decline in earlier stage [7,8,9]. Additional complications between tissue and blood test concordance may arise due to allelic imbalance between regions of the tumor shedding into the bloodstream relative to regions isolated and therefore underrepresented or absent in the blood [9]. Conversely, studies that have compared mutation profiles from tissue obtained at different metastatic sites of the same patient have demonstrated the occurrence of intra-tumoral heterogeneity. Thus, a single tumor biopsy collected from a single location at a single point in time similarly introduces sensitivity concerns and may not represent the tumor mutation profile of that found in circulation [10]. In line with these findings and the increasing use of liquid biopsy within standard clinical practice, diagnostic and therapy developers are including liquid biopsy in clinical studies for patient selection, monitoring of drug response, detection of minimal residual disease (MRD) and for the identification of molecular mechanisms resulting in resistance to therapies [11].

Multiple technologies are used in clinical laboratories for detecting rare variants in cell-free nucleic acid (cfNA) from blood. These technologies are designed to detect targets at low concentration in the circulation, typically less than 1% allele frequency, and have correspondingly low error rates to prevent false positives. Liquid biopsy-based testing includes both Food and Drug Administration (FDA)-approved and Laboratory Developed Tests (LDTs) and utilizes technologies such as digital Polymerase Chain Reaction (dPCR), amplification refractory mutation system (ARMS), allele-specific PCR (AS-PCR) and next-generation sequencing (NGS) [12]. The selection of a technology is usually driven by the clinical question and intended use. In a setting where there are limited variants of interest, for example when looking at a small number of actionable targets associated with approved treatment, approaches that use targeted PCR methods can have benefits including high sensitivity, short turn-around times and lower cost. In contrast, broad-profiling of the cell-free compartment in blood can be obtained with NGS and may support a more comprehensive understanding of the molecular profile of the tumor. The benefit of this broader approach is evident in its extensive adoption for use in clinical research that looks for new drug targets, as well as for clinical trial enrollment [13].

We have deployed a NGS platform for use in clinical trial testing and in new assay development and have previously reported on these efforts [14,15]. This report focuses on performance verification and implementation of a liquid biopsy application in our College of American Pathologists/Clinical Laboratory Improvement Amendments (CAP/CLIA) certified clinical testing laboratory. To eliminate logistic barriers associated with cold-chain specimen management and to broaden access, we have verified the use of an FDA-approved nucleic acid stabilization tube (Streck cell-free DNA Blood Collection Tube^®^) for specimen collection, which allows for the ambient shipment of whole-blood to the centralized laboratory [16,17,18]. Once the specimens arrive at the clinical laboratory, total nucleic acids are isolated from the blood plasma using previously published methods [19]. Reverse transcription is performed using the total nucleic acid eluates without separating cell-free DNA (cfDNA) from cell-free (cfRNA). This provides several advantages including maximizing sample usage and simplifying the workflow. Amplicon-based targeted NGS is performed using the Ion GeneStudio™ S5 Prime, a semiconductor-based system and an Oncomine™ Pan-Cancer Cell-Free Assay [20,21]. Amplicon-based enrichment approaches are well-suited for small, targeted panels, and benefit from lower input requirements and simplified workflows [20,22]. By contrast, large panels and whole-exome sequencing assays may benefit from superior coverage and uniformity that hybridization capture methods may provide [23,24]. The sequencing data is processed and analyzed using the on-board S5 Prime informatics pipeline, as provided by Thermo Fisher Scientific, Inc. The Test Result Report (TRR) is generated using variant information extracted from the sequencing analysis software.

Although this manuscript focuses on the performance verification of variants relevant to NSCLC, the targeted NGS panel can detect alterations in 52 genes and includes the identification of substitutions (including single nucleotide variants (SNV) and multiple nucleotide variants (MNV)), insertions, deletions, copy number variations (CNV) and gene fusions/skipping. We describe analytic sensitivity and specificity, within lab precision, and accuracy using retrospectively banked clinical specimens. All reference specimens were associated with previous variant results generated using a validated and New York State Clinical Laboratory Evaluation Program (NYS-CLEP) approved liquid biopsy test that utilizes droplet digital™ PCR (ddPCR) [19].

## 2. Materials and Methods

### 2.1. Clinical Specimens

Clinical specimen use is considered exempt research under 45 CFR 46.104(d)(4), which is the relevant exemptions section to the Common Rule (45 CFR Section 46).

De-identified remnant clinical specimens were selected based on their mutation status as determined by the GeneStrat^®^ Mutation Test (Biodesix, Inc., Boulder, CO, USA), a targeted genotyping approach based on ddPCR™ and previously described [19]. These specimens were evaluated with targeted assays for Epidermal Growth Factor Receptor (EGFR) L858R, EGFR T790M, EGFR exon 19 deletions (EGFR Del19), GTPase Kirsten ras gene (KRAS) G12X and/or B-Raf Proto-Oncogene, Serine/Threonine Kinase (BRAF) V600E variants from cfDNA. When dilutions were required to target specific allele frequencies, contrived clinical specimens were generated by diluting positive specimens using negative NSCLC specimens collected and stored in an equivalent manner.

### 2.2. Specimen Collection, Nucleic Acid Extraction and Reverse Transcription

Whole blood was collected into Cell-Free DNA Blood Collection Tube^®^ (Streck, La Vista, NE, USA) and shipped to the laboratory at ambient temperature [16,17,18]. Plasma was prepared from whole blood within 48 h of collection as described previously [19]. Extraction was performed using the QIAamp Circulating Nucleic Acid Kit (Qiagen, Hilden, Germany) using up to 5 mL of plasma and eluted in a final volume of 100 μL. Following extraction, total nucleic acid (TNA) was concentrated using the RNA Clean-up and Concentration Micro Elute kit (Norgen, Thorold, Ontario, Canada) and eluted in 25 μL and quantified using the Qubit dsDNA High-Sensitivity Assay Kit (Thermo Fisher Scientific, Waltham, MA, USA). Twenty ng was reverse-transcribed using SuperScript VILO (Thermo Fisher Scientific) with parameters as follows: 42 °C for 30 min, 85 °C for 5 min, 10 °C hold.

### 2.3. Library Preparation, Quantification and Pooling

Library preparation was performed on the reverse transcription reaction using the Oncomine Pan-Cancer Cell-Free Assay (Thermo Fisher Scientific) according to the manufacturer’s instructions. Library quantification was performed using the Ion Library TaqMan Quantitation Kit (Thermo Fisher Scientific) on the LightCycler 96 (Roche Diagnostics, Indianapolis, IN, USA). Libraries were diluted to a final concentration of 50 pM, and eight libraries were pooled at equimolar concentration for chip templating.

### 2.4. Templating and Sequencing

Ion 550™ Chips were templated on the Ion Chef™ instrument (Thermo Fisher Scientific) and sequenced on the Ion GeneStudio™ S5 Prime (Thermo Fisher Scientific) using the Ion 550 Kit-Chef (Thermo Fisher Scientific). Sequencing data was analyzed using Torrent Suite Software version 5.12 and Ion Reporter™ version 5.10 (Thermo Fisher Scientific). Variant calling was performed using the Oncomine™ TagSeq Pan-Cancer Liquid Biopsy w2.1—Single Sample workflow within Ion Reporter.

### 2.5. Quality Control (QC) Metrics

Sample level QC thresholds were applied as follows: ≥10 million total mapped reads, ≥95% coverage uniformity, ≥80% molecular-based uniformity, ≥80 bp mean read length and a library concentration of ≥50 pM. Specimens that did not meet these criteria were not included in the analysis.

A contrived analytic positive control was generated and used to monitor each batch for quality assurance. The analytic positive control is a mixture of fragmented genomic DNA and synthesized gene segments (gBlocks; Integrated DNA Technologies, Coralville, IA, USA) representing EGFR L858R, EGFR T790M, EGFR ∆E746-A750, KRAS G12C and BRAF V600E, designed to mimic cfDNA prepared as described previously [19]. This control was admixed with pooled plasma and processed alongside each batch of clinical specimens, beginning at the extraction step and brought through the entirety of the workflow. Only those batches that contained an analytic positive control testing positive for all EGFR, KRAS, and BRAF variants were considered passing.

## 3. Results

**Figure 1:**
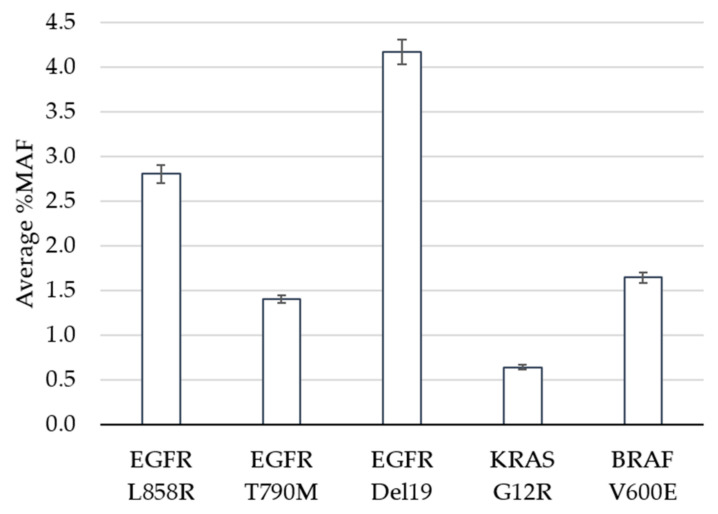
Limit of Detection. The average percent minor allele frequency (%MAF) detected for 5 DNA variants is shown. Error bars indicate ± standard error from the mean for 23 (EGFR Del19) or 24 (EGFR L858R, EGFR T790M, KRAS G12R, BRAF V600E) observations per variant.

The limit of detection study was designed to evaluate performance of the assay at and near the pre-defined cutoff of 0.5% minor allele frequency (MAF). Clinical specimens harboring positive mutations in EGFR, KRAS or BRAF based on the GeneStrat^®^ Mutation Test reference were diluted in negative specimens to generate contrived clinical samples with mean MAFs ranging from 0.6% to 4.2% (Figure 1).

In total, 120 tests were performed (24 replicates each per five variants), 119 of which met QC metrics and were eligible for analysis. The analytic sensitivity, defined as the percentage of times that a variant was detected above the cutoff when expected, was 100% for all variants, including the specimens that contained KRAS at a MAF of 0.6% (Table 1). The percent coefficient of variation (%CV) ranged between 14.4% and 18.7% across the replicates for each variant. The Oncomine™ Pan-Cancer Cell-free NGS assay is designed to detect 982 hotspots in comparison to the targeted approach for the reference ddPCR test which interrogates specific EGFR, KRAS and BRAF mutations. In addition to measuring the reference variant(s), we observed additional mutations within the five contrived clinical specimens (Supplementary Table S1). For example, specimen 1 had 24 positive calls for EGFR L858R (the expected variant based on the reference result) as well as 24 replicates that contained the EGFR L833V and KRAS G13D mutations above the 0.5% MAF cutoff. This highlights the benefit of NGS technology when broader coverage of the genome is of interest.

Sixty clinical specimens were tested to determine the accuracy of the assay. All specimens contained adequate amounts of cfNA to input 20 ng. Of the 60, 7 were removed from the final analysis due to quality control failures. Within the remaining 53 specimens, 6 samples were positive for 2 reference variants each, for a total of 41 positive reference results (8 EGFR Del19, 7 EGFR L858R, 6 EGFR T790M, 6 BRAF V600E and 14 KRAS G12C). Each of the 53 specimens was also evaluated by ddPCR for some or all of the targets for a total of 210 negative reference results (52 EGFR Del19, 52 EGFR L858R, 52 EGFR T790M, 41 BRAF V600E and 33 KRAS G12C). Overall, a total of 251 reference results were used to determine label-level assay agreement (Table 2).

Based on the results for the 251 tests, accuracy, sensitivity and specificity at the variant level were determined (Table 2). True positives are defined as the variants that tested positive by both ddPCR and NGS, and true negatives as variants that tested negative by both ddPCR and NGS. False positives are defined as variants that tested positive by NGS and negative by the reference ddPCR test, and false negatives are variants detected by ddPCR but not NGS.

**Figure 2:**
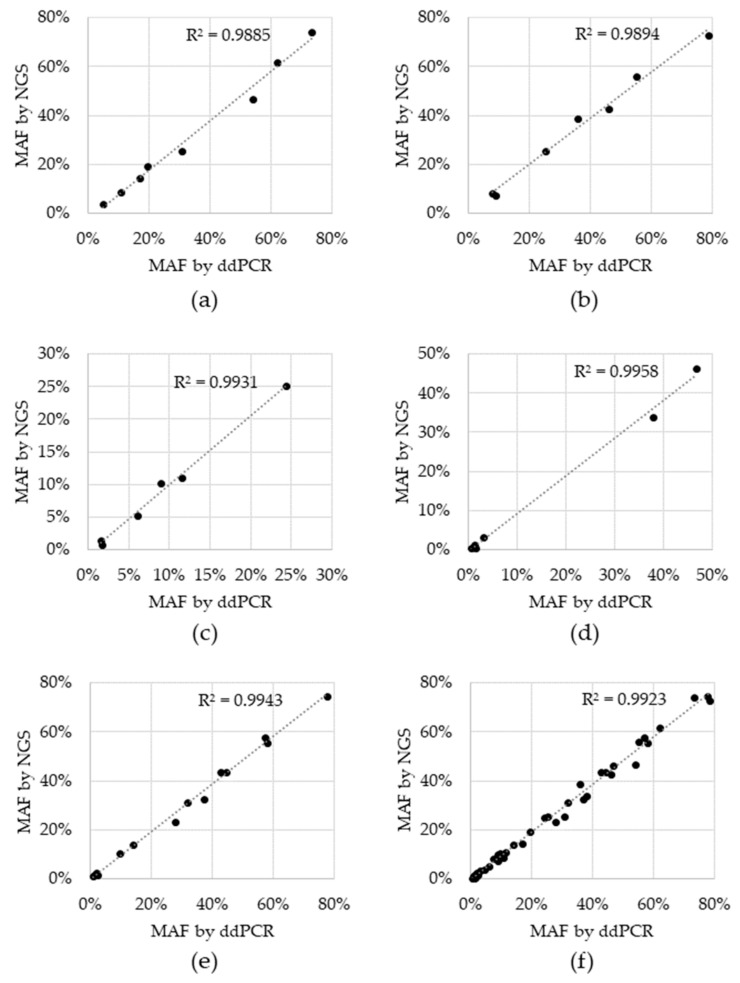
Concordance between Next Generation Sequencing (NGS) and droplet digital Polymerase Chain Reaction (ddPCR) reference test. Black data points represent the percent minor allele detected via NGS (y-axis) and the reference ddPCR MAF result (x-axis) for: (**a**) EGFR del19, (**b**) EGFR L858R, (**c**) EGFR T790M, (**d**) BRAF V600E, (**e**) KRAS G12C and (**f**) all variants combined. Dotted line: linear regression; R^2^: coefficient of determination.

Sensitivity, specificity and accuracy were 100% for all variants, except for BRAF V600E, which was 66.7% sensitive and 95.7% accurate. For the two tests that resulted in a negative call, the BRAF V600E variant was detected, but did not reach the cutoff. These two BRAF V600E discordances had 1.4% and 0.6% MAF via ddPCR and while they were in fact detected by NGS, the %MAF for both fell below the cutoff of 0.5% MAF at 0.42% and 0.38%, respectively (Supplementary Table S2). Concordance was also assessed for allele fraction, which was determined by plotting the %MAF as measured by the reference ddPCR test to that measured by NGS for each positive reference. A linear regression analysis was performed for each individual variant, as well as for all variants (Figure 2). The coefficient of determination (R^2^) for the regression analysis ranged from 0.9885 to 0.9958 for individual variants and was 0.9923 for the entire dataset, indicating that there is high concordance between the two technologies.

**Figure 3:**
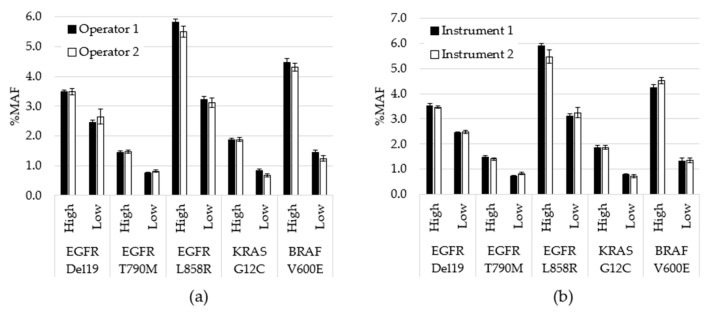
Performance comparison for operators and instruments. (**a**) All data generated by each operator, regardless of day or instrument, was averaged for each variant level (*p* = 0.78). (**b**) All data generated using each instrument, regardless of operator or day, was averaged for each variant level (*p* = 0.99). Error bars indicate ± standard error of the mean. *p*-value calculated using student’s *t*-test.

Assay precision was assessed by testing contrived specimens over multiple days using two operators and two instruments. Contrived specimens were generated so that each variant was tested at two allele frequencies and in duplicate runs: 240 unique tests were performed, 203 were included in the analysis, while 37 were removed due to QC failures. Overall, allele frequencies were consistent over nine days of testing with no significant differences between observed testing conditions (inter-operator *p* = 0.78; inter-instrument *p* = 0.99). To visualize the variability between operators at the variant level on each day of testing, the average %MAF for the 2 replicate tests performed by each operator was plotted (Figure 3). CV was also calculated between variables. For example, for inter-day, the %CV was calculated for each variant based on 9 data points. Each data point was the average for all MAF values for a particular variant for that day (*n* = 4). The cumulative %CV was calculated by averaging all %CVs, and was 8.4% for inter-day, 4.0% for inter-operator and 3.4% for inter-instrument.

## 4. Discussion

Historically, the practice of evaluating genetic biomarkers in patients with solid tumor required an invasive tissue biopsy. However, liquid biopsy provides physicians with a complimentary approach that benefits from a minimally invasive blood-draw [1]. Our goal was to verify the performance of the cell-free NGS panel for use in a regulated clinical laboratory. The described studies were performed with approved standard operating procedures, qualified instruments and reagents and competent personnel, in accordance with good laboratory practices. Although the assay can pick up variants at frequencies as low as 0.1% when using an input of 20 ng, in this report, we took a more conservative approach to validation using the 0.5% allele frequency cutoff for SNV and indels to differentiate positive from negative results.

Three studies are described including limit of detection, accuracy and precision. Clinical specimens were selected at and near the assay cutoff for limit of detection evaluation. Of variants, 100%, including the specimens that contained KRAS at an MAF of 0.6%, were detected in all replicates. The coefficient of variation was below 20% for all positive variants, ranging between 14.4% and 18.7%, even at the low MAF values, demonstrating consistency of the NGS assay at the lower boundary. A within-lab precision study further evaluated variability between replicate runs of the same specimens, specifically assessing the differences in operators, instruments and between days of testing. A comparison of the allele frequencies of the positive mutations confirmed no significance between results generated on different instruments or with different operators. Additionally, the CV was less than 10% in each case (inter-day of 8.4%, inter-operator of 4.0% and inter-instrument of 3.4%).

Furthermore, results were consistent between the NGS assay when compared to the orthogonal NYS-CLEP-approved ddPCR workflow when evaluated using 53 clinical specimens. When directly comparing the allele frequencies, we observed a strong linear correlation between NGS and ddPCR methods. Mutations were also compared in a pairwise analysis based on their positive or negative result by each test method. We found 100% negative agreement demonstrating a high level of assay specificity. Likewise, all three EGFR variants and KRAS demonstrated 100% positive agreement. BRAF V600E might be considered the underperformer, whereby we detected 66.7% (4/6) of the specimens with a positive BRAF V600E reference ddPCR result using the NGS assay. Although this may be due to specific inefficiencies in the amplicon design or bioinformatics pipeline, it is also likely due to the differences observed at low %MAF more generally. Of note, the two false negative clinical specimens had 1.4% and 0.6% MAF via ddPCR, and while negative by NGS, these two samples did have detectable BRAF V600E molecular reads.

Our results align well with available liquid biopsy NGS tests for use in molecular diagnosis and monitoring. According to a recent cross-test study that evaluated four on-market liquid biopsy NGS tests, false negatives were observed at or below 1% variant frequencies [18]. Our results were similar, whereby both assays picked up some BRAF V600E signal, but for NGS, the allele frequencies were below the cut-off of the assay. The differences in these tests when specimens have very low level of circulating tumor nucleic acid can be due to stochastic sample biases, assay design, thresholding nuances or might be a combination of the former [19].

In addition to identifying those actionable variants for which we had ddPCR orthogonal reference results and that were used for evaluation of NGS test performance, we also identified nonsynonymous variants of unknown significance (VUS) in our clinical samples. VUS are common both in EGFR and many other regions of the genome. For example, we found cooccurrence of EGFR L833V and KRAS G13D mutations along with EGFR L858R (Supplementary Table S1) in one of the specimens evaluated. While neither of the former variants are currently approved biomarkers in NSCLC, functional evaluation of co-occurring VUS within EGFR may alter response to target tyrosine kinase inhibition [20]. This result highlights a benefit of NGS technology when broader coverage of the genome is of interest for clinical research. However, VUS must be carefully evaluated. In many cases, these mutations can be attributed to DNA shed from normal cells, including germline variants or noncancerous somatic variants that evolve from the process of clonal hematopoiesis. Altogether, our results align with the biological and technical challenges that the NGS field faces as we push this liquid biopsy technology to detect low levels of circulating tumor, which is especially critical in earlier stages of disease [19] or even more so as we enter into cancer screening.

Implementation of NGS-based testing in the clinic has highlighted the importance and potential benefits for oncology. However, standardizing the genetic interpretation and reporting of the complex molecular results such as VUS and co-occurring mutations among laboratories is critical. Guidelines to report and interpret NGS variants have been published by clinical societies to help guide the community [25]. These guidelines highlight the importance of evidence-based variant annotation and tier-based categorization supported by curated databases, such as the Oncomine™ Knowledgebase Reporter, which are regularly maintained to align with current clinical evidence. Each case should first be reviewed by a trained molecular diagnostic professional prior to releasing the NGS report. Ultimately, patient management decisions are made by the physician and are based on genetic alterations along with the other medical information associated with the case. Increasingly, assay development efforts have focused on screening and early detection. In these settings, panels that detect the common somatic mutations associated with tumorigenesis, like the one described here, will not be sufficient on their own, but recent data suggests they can be improved by combining their results with other biomarkers and clinical information. For example, both DNA methylation and fragmentation signals obtained from sequencing data have been demonstrated to increase the percentage of cancer cases identified for cancer screening [21,22]. In addition to improving sensitivity, these tests also have the challenges of accurately identifying the correct origin of the cancer, which can be a difficult problem when the biomarker is found in circulation. Recent progress has been made by combining circulating tumor DNA markers with protein signatures and Positron Emission Tomography-Computed Tomography (PET-CT) imaging to this end [23].

## 5. Conclusions

Genotyping of liquid biopsy using NGS technology is suitable for implementation in routine clinical decision-making for advanced NSCLC patients. We have demonstrated that the assay is capable of rapidly detecting SNVs and deletions with the performance needed to meet clinical testing requirements using the EGFR, BRAF and KRAS genes. We intend to extend our validation studies to additional genes and variants including CNV and fusion/skipping, for which the current assay design is capable using cfDNA and cfRNA, respectively. Liquid biopsy tests such as the one described can clearly detect actionable genomic alterations in patients who are unable to undergo biopsies, whose biopsies have not yielded sufficient, or material of sufficient quality for molecular testing. Liquid biopsy tests have the added benefit of not being partial to biopsy location as can be a confounding issue with tissue heterogeneity. The combination of the two rapid approaches for analyzing cell-free nucleic acids—the targeted, low-cost ddPCR-based genotyping assays, and the broader NGS method—provide complementary ways in which to investigate the molecular characteristics of each NSCLC tumor.

## Figures and Tables

**Table 1:** Analytic sensitivity near assay cutoff.

Variant	Expected	Detected	Hit Rate
EGFR L858R	24	24	100%
EGFR T790M	24	24	100%
EGFR E746-A750	23	23	100%
BRAF V600E	24	24	100%
KRAS G12R	24	24	100%

**Table 2:** Clinical accuracy results.

Variant	TP ^1^	TN ^2^	FP ^3^	FN ^4^	Total	Accuracy	Sensitivity	Specificity
EGFR Del19	8	45	0	0	53	100.0%	100.0%	100.0%
EGFR L858R	7	45	0	0	52	100.0%	100.0%	100.0%
EGFR T790M	6	46	0	0	52	100.0%	100.0%	100.0%
BRAF V600E	4	41	0	2	47	95.7%	66.7%	100.0%
KRAS G12C	14	33	0	0	47	100.0%	100.0%	100.0%

^1^ TP: True Positive, ^2^ TN: True Negative, ^3^ FP: False Positive, ^4^ FN: False Negative.

## Data Availability

The authors confirm that the data supporting the findings of this study are available within the article and its supplementary materials.

## Supplementary Materials

The following are available online at https://www.mdpi.com/2075-4418/11/2/155/s1, Table S1: All variants within limit of detection study detected above the 5% MAF cutoff, Table S2: Clinical accuracy results for positive samples.
