# Supplementary material for: Targeted Next-Generation Sequencing of Liquid Biopsy Samples from Patients with NSCLC
Source: Diagnostics (Basel). 2021 Jan 21;11(2):155. doi: 10.3390/diagnostics11020155 (PMC7912015; doi:10.3390/diagnostics11020155)
Supplement: Supplementary file 1 [file diagnostics-11-00155-s001.pdf]

Table S1. All variants within limit of detection study detected above the 5% MAF cutoff.

Specimen	Detected Variant	ddPCR Reference	# Replicates Detected/Total Replicates Performed
**1**	EGFR L858R	Positive	24/24
	EGFR L833V	Not Tested	24/24
	KRAS G13D	Not Tested	24/24
	TP53 Y220C	Not Tested	2/24
**2**	EGFR T790M	Positive	24/24
	EGFR L858R	Positive	24/24
	TP53 Y220C	Not Tested	1/24
**3**	EGFR E746-A750	Positive	23/23
	TP53 V272L	Not Tested	23/23
	RAF1 S257L	Not Tested	1/23
**4**	KRAS G12R	Positive	24/24
	FGFR3 F384L	Not Tested	24/24
	TP53 Y220C	Not Tested	24/24
**5**	BRAF V600E	Positive	24/24
	TP53 Y220C	Not Tested	24/24

Table S2. Clinical Accuracy Results for Positive Samples

Gene Variant	Detected Variant	%MAF by ddPCR	ddPCR Result	%MAF by NGS	Result	Label Agreement
BRAF V600E	p.V600E	37.9%	Positive	33.99%	Positive	Yes
	p.V600E	3.0%	Positive	3.31%	Positive	Yes
	p.V600E	1.4%	Positive	0.42%	Negative	No
	p.V600E	1.2%	Positive	1.17%	Positive	Yes
	p.V600E	0.6%	Positive	0.38%	Negative	No
	p.V600E	46.70%	Positive	46.38%	Positive	Yes
EGFR Del19	p.E746_A750del	62.00%	Positive	61.70%	Positive	Yes
	p.E746_A750del	53.91%	Positive	46.68%	Positive	Yes
	p.E746_A750del	30.66%	Positive	25.44%	Positive	Yes
	p.E746_A750del	19.37%	Positive	19.12%	Positive	Yes
	p.E746_A750del	4.70%	Positive	3.95%	Positive	Yes
	p.E746_A750del	17.03%	Positive	14.40%	Positive	Yes
	p.L747_P753delinsS	73.23%	Positive	74.24%	Positive	Yes
	p.L747_T751del	10.72%	Positive	8.59%	Positive	Yes
EGFR L858R	p.L858R	78.56%	Positive	72.85%	Positive	Yes
	p.L858R	55.14%	Positive	55.89%	Positive	Yes
	p.L858R	45.87%	Positive	42.59%	Positive	Yes
	p.L858R	35.86%	Positive	38.92%	Positive	Yes
	p.L858R	25.16%	Positive	25.63%	Positive	Yes

	p.L858R	7.65%	Positive	8.35%	Positive	Yes
	p.L858R	8.99%	Positive	7.49%	Positive	Yes
EGFR T790M	p.T790M	24.38%	Positive	25.09%	Positive	Yes
	p.T790M	11.56%	Positive	11.07%	Positive	Yes
	p.T790M	8.96%	Positive	10.15%	Positive	Yes
	p.T790M	1.53%	Positive	1.48%	Positive	Yes
	p.T790M	1.63%	Positive	0.69%	Positive	Yes
	p.T790M	6.12%	Positive	5.20%	Positive	Yes
KRAS G12C	p.G12C	77.79%	Positive	74.34%	Positive	Yes
	p.G12C	58.04%	Positive	55.67%	Positive	Yes
	p.G12C	57.08%	Positive	57.91%	Positive	Yes
	p.G12C	44.37%	Positive	43.61%	Positive	Yes
	p.G12C	42.76%	Positive	43.79%	Positive	Yes
	p.G12C	37.10%	Positive	32.50%	Positive	Yes
	p.G12C	31.86%	Positive	31.37%	Positive	Yes
	p.G12C	27.79%	Positive	23.12%	Positive	Yes
	p.G12C	14.05%	Positive	14.03%	Positive	Yes
	p.G12C	10.17%	Positive	QC failure	NA	NA
	p.G12C	9.79%	Positive	10.63%	Positive	Yes
	p.G12C	8.41%	Positive	QC failure	NA	NA
	p.G12C	8.12%	Positive	QC failure	NA	NA
	p.G12C	6.37%	Positive	QC failure	NA	NA
	p.G12C	2.49%	Positive	1.52%	Positive	Yes
	p.G12C	2.07%	Positive	2.46%	Positive	Yes
	p.G12C	1.88%	Positive	QC failure	NA	NA
	p.G12C	1.37%	Positive	1.46%	Positive	Yes
	p.G12C	0.85%	Positive	1.33%	Positive	Yes
